# UNC-52 localization with respect to longitudinal axon tracts in the ventral and dorsal nerve cords in *C. elegans*

**DOI:** 10.17912/micropub.biology.001703

**Published:** 2025-07-18

**Authors:** Debapriya Roy, Harald Hutter

**Affiliations:** 1 Biological Sciences, Simon Fraser University

## Abstract

UNC-52 /Perlecan is a core basement membrane (BM) component essential for connecting muscle cells to overlying hypodermal cells. In the nervous system of *C. elegans* , *unc-52 ( ra515 )* causes dendritic branching defects in the PVD neuron independent of muscle attachment defects. We found that *unc-52 ( ra515 )* also causes axon navigation defects in the ventral nerve cord (VNC). We then examined the localization of UNC-52 with respect to ventral and dorsal nerve cord (DNC) axon tracts. We found that UNC-52 is localized underneath muscle cells as previously observed but is absent from BMs surrounding ventral and dorsal axon tracts at all stages of development.

**Figure 1. UNC-52 localization with respect to ventral and dorsal nerve cords:**
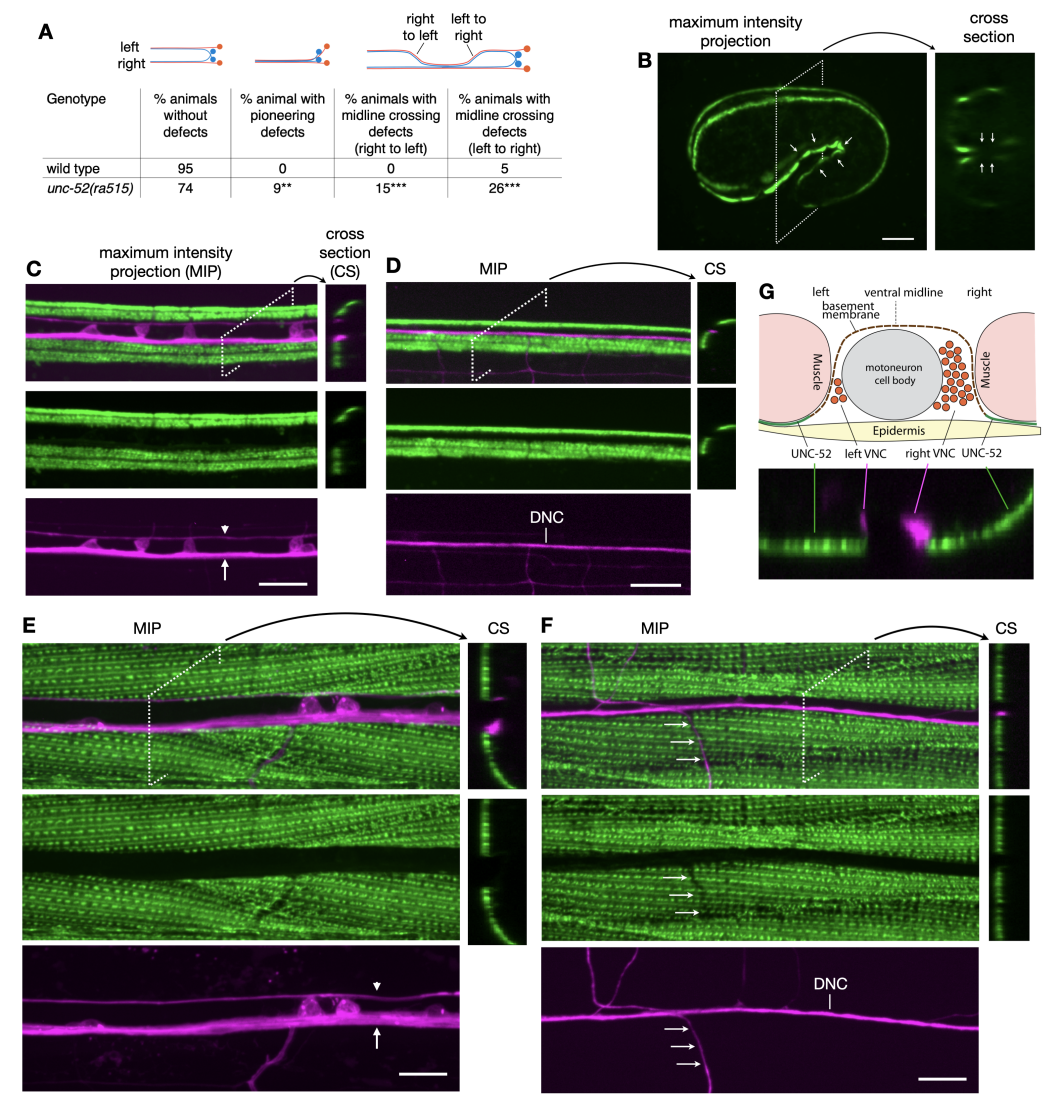
(A) PVP and PVQ axon navigation defects in *unc-52 ( ra515 )* . Some animals have multiple defects. *** P < 0.001, ** P < 0.01 (χ2 test). n = 100. Marker used: *hdIs26* [ *odr-2 ::CFP, sra-6 ::DsRed2* ]. (B,C,D,E,F) UNC-52 :mNG expression in dorsal and ventral nerve cords. UNC-52 ::mNG ( *qy80* ) is shown in green and the panneuronal marker *; hdIs36* [ *rgef-1 ::DsRed2* ] is shown in magenta. The location of the cross-sections is indicated by a rectangle. Images are maximum intensity projections. Anterior is to the left. Scale bar is 10μm. (B) 1 1/2-fold embryo, side view and cross-section. The panneuronal marker is not expressed at this stage. Arrows point to the VNC. (C) young larva (~L2 stage), VNC. (D) young larva(~L2 stage), DNC. (E) adult, VNC. (F) adult, DNC. Arrows in panels C and E point to the right axon tract, arrowheads to the left axon tract. Arrows in panel F point to one of the commissures. (G) Schematic cross-section of the VNC, illustrating the localization of UNC-52 with respect to VNC axon tracts and the basement membrane surrounding the axon tracts. The cross-section from panel E is shown below the drawing for comparison.

## Description

The basement membrane (BM) is a sheet of cross-linked secreted proteins that provides structural support for tissues and an adhesive substrate for migrating cells and outgrowing neuronal processes such as axons (see Sherwood, 2021; Töpfer, 2023; Walma and Yamada, 2020 for recent reviews). UNC-52 /Perlecan is one of the core components of the BM (Rogalski et al., 1993). In *C. elegans ,* UNC-52 is secreted from muscle cells and localizes to dense bodies and M-lines underneath muscle cells (Francis and Waterston, 1991; Mullen et al., 1999). It is essential for myofilament assembly in the muscle, attachment of the myofilaments to the cell membrane and connecting muscle cells to overlying hypodermal cells (Gilchrist and Moerman, 1992; Mackenzie et al., 1978). In the developing nervous system, UNC-52 is involved in axon navigation and dendrite patterning. We identified an *unc-52* allele, *hd133* , in a genetic screen for axon guidance mutants. *unc-52 ( hd133 )* mutants have weakly penetrant midline crossing defects in the ventral nerve cord, VNC (Taylor and Hutter, 2019). *unc-52 ( hd133 )* is a missense mutation in a laminin EGF domain and does not obviously affect muscle attachments. How it affects axon navigation is currently unclear. The PVD neuron forms a regular pattern of dendritic branches between muscle and hypodermis. The pattern of the terminal branches is dependent on regularly spaced stripes of the adhesion molecule SAX-7 /L1CAM on the hypodermal cells. The spacing of SAX-7 is established by the regular pattern of UNC-52 at the sarcomere dense bodies, where it also provides a link to the hemidesmosome on the hypodermal cells. Consequently, mutations in *unc-52* lead to patterning defects of PVD dendritic branches (Liang et al., 2015). A later study found a second role for UNC-52 in PVD dendrite patterning that is independent of its role in muscle-hypodermis attachment (Celestrin et al., 2018). The *unc-52 ( ra515 )* allele is an in-frame deletion removing four immunoglobulin domains that are required for the binding of NID-1 /Nidogen, another core basement membrane component. NID-1 is mislocalized in *unc-52 ( ra515 )* mutants leading to dendritic patterning defects.

Here we tested whether *unc-52 ( ra515 )* affects axon navigation in the VNC focussing on PVP and PVQ axons in the left and right axon tracts. We found two weakly penetrant defects in PVPR pioneer navigation in *unc-52 ( ra515 )* mutant animals ( Figure 1A ). First, in a small number of animals (9%) the PVPR axon fails to pioneer the left VNC axon tract and initially extends in the right axon tract together with the follower PVQL axon. In all these animals the PVPR and PVQL axons eventually crossed the midline to establish the left tract, typically before they reached the vulva region. Second, in a larger number of animals the PVP and PVQ axons inappropriately cross the ventral midline to extend in the contralateral axon tract. In 26% of the animals, axons cross from the left into the right axon tract. In 15% of these animals the axons eventually cross back into the left axon tract (right to left cross-over).

To determine how *unc-52* might affect VNC axon navigation, we examined the localization of UNC-52 with respect to the VNC axon tracts. We generated a strain that expresses mNeonGreen tagged UNC-52 ( UNC-52 ::mNG) and a panneuronal DsRed marker to label neurons and their axons. We found that UNC-52 is expressed underneath muscle cells at dense bodies as previously described (Francis, 1991; Mullen, 1999). Unexpectedly, we discovered that UNC-52 is not present in BMs surrounding VNC axon tracts. In embryos, the panneuronal marker is not yet expressed but the position of the VNC can be deduced from the orientation of the embryo. UNC-52 expression at the 1 1/2-fold stage, the time of pioneer axon outgrowth, is consistent with its absence in the VNC ( Figure 1B ). Neither young larvae ( Figure 1C ) nor adults ( Figure 1E ) show UNC-52 expression in the VNC. UNC-52 is also not present in the dorsal nerve cord, DNC ( Figure 1D, F). We noticed that UNC-52 expression at dense bodies is reduced or absent in locations where commissures have grown from the ventral to the dorsal nerve cord (arrows in Figure 1F ), suggesting that outgrowing commissures can locally disrupt contacts between muscle and hypodermis. Figure 1G provides a schematic of the observed UNC-52 expression with respect to VNC axon tracts.

Several mutations in different parts of UNC-52 cause VNC navigation defects. *unc-52* produces 16 different splice variants. The mNeonGreen tag is located close to the C-terminus and will tag 14 of the 16 splice variants (Keeley et al., 2020) including all those containing the four IG domains deleted in *ra515* . Therefore, the UNC-52 ::mNG expression should reflect the localization of the splice variants involved in VNC axon navigation. We expected UNC-52 to be localized in the basement membrane surrounding VNC axon tracts. However, this does not seem to be the case, which leaves the role of UNC-52 in VNC axon navigation unclear at this point. It is possible that small amounts of UNC-52 are present but are below the detection limit of our microscope. It is also possible that UNC-52 localization itself is changed in the *unc-52* alleles that affect axon navigation and that mislocalized UNC-52 causes axon navigation defects. Addressing this question requires the introduction of the relevant mutations by CRISPR/Cas9 in the UNC-52 ::mNG strain, which we consider to be outside the scope of this study. Further experiments will be required to explain the role of *unc-52* in VNC axon navigation.

## Methods

**Phenotypic analysis of axon guidance defects and microscopy**

For phenotypic analysis of axonal defects adult animals from a growing population (20˚ C) were immobilized with 10 mM sodium azide and mounted on 2% agarose pads. Axonal defects were scored with a Zeiss Axioscope (40× objective).

For the analysis of UNC-52 ::mNG expression animals were imaged on a Zeiss Axioplan II microscope equipped with a Quorum WaveFX spinning disc system (Quorum Technologies, Canada). Stacks of confocal images with 0.1 to 0.2 μm distance between focal planes were recorded. Volocity software (Quorum Technologies, Canada) was used for image acquisition and analysis. Images in the figure are maximum intensity projections of all focal planes.

## Reagents

**Table:** 

Strain	Genotype	Source
VH648	*hdIs26* [ *odr-2 ::CFP, sra-6 ::DsRed2* ] III	our lab
VH3039	*unc-52 ( ra515 )* II *; hdIs26* [ *odr-2 ::CFP, sra-6 ::DsRed2* ] III	(Mullen et al., 1999) and our lab
VH3016	*unc-52 ( qy80* [ *mNG+loxP (synthetic exon):: unc-52* ] *)* II *; hdIs36* [ *rgef-1 ::DsRed2* ] IV	(Keeley et al., 2020) and our lab
